# m6A reader IGF2BP2 promotes M2 macrophage polarization and malignant biological behavior of bladder cancer by stabilizing NRP1 mRNA expression

**DOI:** 10.1186/s12894-024-01534-4

**Published:** 2024-07-16

**Authors:** Dian Fu, Xiuquan Shi, Xiaoming Yi, Ding Wu, Haowei He, Wenquan Zhou, Wen Cheng

**Affiliations:** 1 Department of Urology, Jinling College of Clinical Medicine, Nanjing Medical University, No.305, Zhongshandong Road, Xuanwu District, Nanjing, Jiangsu 210002 China; 2 Department of Urology, Nanjing Jinling Hospital, Affiliated Hospital of Medical School, Nanjing University, No.305, Zhongshandong Road, Xuanwu District, Nanjing, Jiangsu 210002 China

**Keywords:** Bladder cancer, IGF2BP2, NRP1, M2 macrophage polarization

## Abstract

**Background:**

Insulin-like growth factor 2 mRNA-binding protein 2 (IGF2BP2) has been confirmed to play oncogenic role in many cancers. However, the role and mechanism of IGF2BP2 in bladder cancer (BCa) still deserves to be further revealed.

**Methods:**

The mRNA and protein levels of IGF2BP2 and neuronilin-1 (NRP1) were detected by real-time quantitative PCR (RT-qPCR) and western blot. Cell proliferation, apoptosis, migration and invasion were determined using colony formation assay, EdU assay, CCK8 assay, flow cytometry and transwell assay. Xenograft tumor model was conducted to evaluate the role of IGF2BP2 in vivo. THP-1-M0 macrophages were co-cultured with the condition medium (CM) of BCa cells to induce polarization. M2 macrophage polarization was assessed by detecting the mRNA levels of M2 macrophage markers using RT-qPCR and measuring the proportion of M2 macrophage markers using flow cytometry. Moreover, MeRIP and RIP assay were performed to assess m6A level and the interaction between IGF2BP2 and NRP1.

**Results:**

IGF2BP2 and NRP1 were upregulated in BCa tissues and cells. IGF2BP2 knockdown suppressed BCa cell growth and metastasis, as well as inhibited BCa tumor growth. After THP-1-M0 macrophages were co-cultured with the CM of BCa cells, the levels of M2 macrophage markers were markedly enhanced, while this effect was abolished by IGF2BP2 knockdown. IGF2BP2 level was positively correlated with NRP1 level, and it could increase NRP1 mRNA stability. NRP1 overexpression reversed the suppressive effect of IGF2BP2 knockdown on M2 macrophage polarization and BCa cell progression.

**Conclusion:**

m6A-reader IGF2BP2 enhanced M2 macrophage polarization and BCa cell progression by promoting NRP1 mRNA stability.

**Supplementary Information:**

The online version contains supplementary material available at 10.1186/s12894-024-01534-4.

## Highlights

IGF2BP2 knockdown inhibits BCa cell growth and metastasis;

Interference of IGF2BP2 reduces BCa tumor growth;

IGF2BP2 promotes M2 macrophage polarization;

IGF2BP2 enhances NRP1 mRNA stability by m6A modification;

NRP1 silencing represses M2 macrophage polarization and BCa progression.

**Supplementary Information:**

The online version contains supplementary material available at 10.1186/s12894-024-01534-4.

## Introduction

Bladder cancer (BCa) is a common urologic malignancy with high morbidity and mortality [1, 2]. Despite the effectiveness of radiotherapy and radical surgery, patients with BCa have a poor 5-year survival rate because most patients have distant metastases at the time of diagnosis [3, 4]. Therefore, in-depth study of the underlying mechanisms of BCa process is important to develop its therapeutic strategies. Many studies have shown that macrophage polarization is related to the malignant process of BCa, especially M2 macrophage polarization can promote BCa progression [5–7]. Exploring the molecular mechanisms that affect M2 macrophage polarization may also provide new ideas for the pathological process of BCa.

Insulin-like growth factor 2 mRNA-binding protein 2 (IGF2BP2), an RNA-binding protein (RBP), plays an important role in cell growth and human disease progression [8, 9]. As an N6-methyladenosine (m6A) reader, IGF2BP2 is involved in cancer progression by binding to different RNAs [10, 11]. For example, IGF2BP2 enhanced ovarian cancer cell growth and metastasis via promoting CKAP2L translation by m6A modification [12]. IGF2BP2 could increase HMGA1 mRNA stability, thereby accelerating gastric cancer cell metastasis [13]. Wu et al. screened and identified 12 RBPs including IGF2BP2 that might predict the prognosis of BCa patients [14]. Besides, Zhao et al. reported that lncRNA AGAP1-AS1 accelerated BCa proliferation and metastasis via recruiting IGFBP2 protein to increase LRG1 mRNA stability [15]. Importantly, IGF2BP2 recruitment has been shown to promote mRNA stability, which in turn promotes M2 macrophage polarization to accelerate cancer malignant progression [16, 17]. Through database analysis, we found that IGF2BP2 was overexpressed in BCa tissues and was associated with poor prognosis of BCa patients. However, whether IGF2BP2 affects M2 macrophage polarization and BCa progression remains unclear.

Neuronilin-1 (NRP1), a non-tyrosine kinase transmembrane glycoprotein, is known to be a receptor for both the SEMA3 family and the VEGF family [18, 19]. NRP1 is highly expressed in many tumor tissues and participates in cell angiogenesis, growth and metastasis [20, 21]. High NRP1 expression induced M2 macrophage polarization and promoted colorectal cancer metastasis [22]. Previous studies had suggested that high NRP1 level was closely associated with poor prognosis and M2 macrophage polarization in muscle-invasive BCa patients [23]. Also, suppressing NRP1 protein expression could suppress the tumorigenesis of BCa [24]. In this, we found that IGF2BP2 could positively regulate NRP1 protein expression, and NRP1 mRNA had 2 m6A binding sites. However, whether IGF2BP2 mediates M2 macrophage polarization and BCa progression by regulating NRP1 has not been investigated.

Here, we aimed to reveal IGF2BP2 roles and underlying molecular mechanisms in M2 macrophage polarization and BCa progression. Based on the above, we hypothesized that IGF2BP2 regulated NRP1 stability through m6A modification, thereby mediating M2 macrophage polarization and malignant progression of BCa.

## Materials and methods

### Samples

A total of 55 BCa tumor tissues and paired adjacent normal tissues were obtained from 55 BCa patients at Jinling College of Clinical Medicine, Nanjing Medical University. Inclusion criteria: diagnosed as BCa through pathological examination, not treated with surgery and chemoradiotherapy prior to admission. Exclusion criteria: patients with other diseases or a history of treatment for other cancers. All samples were stored at -80 °C. Each patient signed written informed consent, and this study was approved by the Ethics Committee of Jinling College of Clinical Medicine, Nanjing Medical University.

### Cell culture and transfection

Human ureteral epithelial immortalized cells (SV-HUC-1), BCa cell lines (T24 and 5637), and monocytic leukemia (THP-1) were purchased from Procell (Wuhan, China). BCa cell lines and THP-1 cells were cultured in RPMI-1640 medium (Gibco, Grand Island, NY, USA), while SV-HUC-1 cells were cultured in Ham’s F-12 K medium (Gibco). All medium plus 10% FBS (Gibco), 1% P/S (Procell) and additional 0.05mM β-mercaptoethanol for THP-1 cells. THP-1 cells were cultured with 100 ng/mL PMA (Solarbio, Beijing, China) for 24 h to induce THP-1-M0 macrophages.

T24 and 5637 cells were transfected with the shRNA and overexpression vector of IGF2BP2/NRP1/METTL3 (sh-IGF2BP2/OE-IGF2BP2, sh-NRP1/OE-NRP1 and sh-METTL3) using Lipofectamine 3000 (Invitrogen, Carlsbad, CA, USA).

### Co-culture system

Transfected T24 and 5637 cells were seeded in 15-cm culture plates (2 × 10^7^ cells) and cultured for 48 h. The conditioned media (CM) were collected, and THP-1-M0 macrophages were co-cultured with CM for 24 h.

### Real-time quantitative PCR (RT-qPCR)

Total RNA was extracted by TRIzol reagent (Invitrogen). After reverse-transcribed into cDNA, PCR was conducted using SYBR Green (Takara, Tokyo, Japan) and specific primers (Table 1). Relative mRNA level was evaluated by the 2^−ΔΔCt^ method.

**Table 1:** Primer sequences used for RT-qPCR

Name		Primers for PCR (5’-3’)
NRP1	Forward	GGCGCTTTTCGCAACGATAAA
	Reverse	TCGCATTTTTCACTTGGGTGAT
IGF2BP2	Forward	ATGTGGAACAAGTCAACACAGACA
	Reverse	TCTCAAACTGATGCCCGCTT
CD206 (MRC1)	Forward	GCCTCGTTGTTTTGCGTCTT
	Reverse	GAGAACAGCACCCGGAATGA
Arginase-1	Forward	TTCACACCAGCTACTGGCAC
	Reverse	CCCAGGGATGGGTTCACTTC
IL-10	Forward	TTGCCTGGTCCTCCTGACTG
	Reverse	TCACTCTGCTGAAGGCATCTC
TGF-β	Forward	GGAAATTGAGGGCTTTCGCC
	Reverse	CCGGTAGTGAACCCGTTGAT
β-actin	Forward	CTTCGCGGGCGACGAT
	Reverse	CCACATAGGAATCCTTCTGACC

### Western blot (WB) analysis

Proteins were isolated by RIPA buffer (Beyotime, Shanghai, China) and quantified by BCA Assay Kit (Beyotime). Samples were separated by 10% SDS-PAGE gels and transferred to PVDF membranes. After incubated with primary antibodies and secondary antibody, membranes were treated with ECL reagent for observing protein signals. The gray value was analyzed by ImageJ software. The antibodies including anti-IGF2BP2 (1:2000, 11601-1-AP, Proteintech, Rosemont, IL, USA), anti-NRP1 (1:5000, 60067-1-Ig), anti-β-actin (1:20000, 66009-1-Ig), Goat anti-Mouse IgG (1:10000, SA00001-1), and Goat anti-Rabbit IgG (1:10000, SA00001-2). We are not able to provide an image of the full-length membrane for the time being since we have cropped the membrane prior to hybridization with the antibody according to the different protein size. We have provided original images of all blots as well as reproduced images as Supplementary files.

### Colony formation assay

T24 and 5637 cells were cultured in 12-well plates (250 cells/well) for 2 weeks. Then, cells were stained with crystal violet for 10 min after fixed with 4% paraformaldehyde. Colony number was counted under a microscope.

### EdU assay

T24 and 5637 cells were seeded in 96-well plates (1 × 10^5^ cells/well) and labelled with 10 µM EdU solution for 2 h and 1 mL DAPI solution for 10 min using EdU Cell Proliferation Kit (Beyotime). Fluorescence signals were observed under a microscope to analyze EdU^+^ cell rate using ImageJ software.

### CCK8 assay

At each time point (24, 48, and 72 h), T24 and 5637 cells were treated with 10 µL CCK8 reagent (Beyotime) for 2 h in 96-well plates (2 × 10^3^ cells/well). Cell viability was determined via testing OD value using a microplate reader at 450 nm.

### Flow cytometry

For detecting cell apoptosis, T24 and 5637 cells were harvested (5 × 10^5^ cells) and then stained with 5 µL Annexin V-FITC and 5 µL PI for 15 min, followed by assessing cell apoptosis rate using BD FACS Calibur flow cytometry with CellQuest Pro software.

T24 and 5637 cell suspensions (5 × 10^5^ cells) were stained with FITC-labeled anti-CD11b (ab269333, Abcam, Cambridge, CA, USA) and PE-labeled anti-CD163 (ab95613, Abcam). After stained with 7AAD (Beyotime), the proportions of CD11b and CD163^+^ were assessed under flow cytometry with CellQuest Pro software.

### Transwell assay

Transwell chambers pre-coated with or without Matrigel (Corning Inc., Corning, NY, USA) were used to evaluate cell invasion (2 × 10^5^ cells/well) and migration (1 × 10^5^ cells/well), respectively. T24 and 5637 cells suspended with RMPI-1640 medium were seeded into the upper chamber. Then, completed medium were filled in the lower chamber. After 24 h, invasion and migration cells were counted under a microscope with ImageJ software after fixation and staining. For THP-1-M0 macrophages co-culture system, THP-1-M0 macrophages (2 × 10^5^ cells/well) were seeded into the upper chamber, and the CM of transfected T24 and 5637 cells was filled into the lower chamber. Then, migrated macrophages were fixed and stained, followed by counted under a microscope with ImageJ software.

### Mice xenograft models

For lentiviral transduction, 5637 cells were infected with lentiviruses sh-NC/sh-IGF2BP2 with 8 mg/ mL of polybrene. After that, stable 5637 cells were selected by 2 µg/mL of puromycin for 72 h. 5637 cells (2 × 10^6^) stably infected with sh-NC/sh-IGF2BP2 were injected subcutaneously into BALB/c nude mice (purchased commercially from Vital River, Beijing, China) (*n* = 5/group). Tumor volume was assessed every 5 days. After 30 days, mice were sacrificed and tumors were harvested from weighting. In addition, a portion of the tissue was used to prepare paraffin sections and then incubated with anti-IGF2BP2 (1:500, 11601-1-AP), anti-Ki-67 (1:200, 27309-1-AP), and anti-E-cadherin (1:5000, 20874-1-AP) according to the instructions of SP kit (Solarbio) for immunohistochemical (IHC) staining. Animal experiments were approved by the Animal Ethics Committee of Jinling College of Clinical Medicine, Nanjing Medical University.

### MeRIP assay

Total RNAs from T24 and 5637 cells were fragmented and incubated with anti-m6A/anti-IgG and Magnetic Beads (Millipore, Billerica, MA, USA). Then, immunoprecipitated RNA was eluted and purified, and NRP1 mRNA enrichment was analyzed by RT-qPCR.

### RIP assay

T24 and 5637 cells transfected with sh-NC/sh-METTL3 were lysed by RIP lysis buffer. Cell lysates were incubated with anti-IgG/anti-IGF2BP2 pre-coated with protein A/G magnetic beads (Millipore). Then, bound RNA was eluted and then used for RT-qPCR to measure NRP1 mRNA enrichment.

### mRNA stability assay

T24 and 5637 cells were treated with Actinomycin D (5 µg/mL, Millipore) for 0, 4 and 8 h. At each time point, cells were harvested for PCR extraction and RT-qPCR to detect NRP1 mRNA level.

### Statistical analysis

Data are presented as the mean ± SD. Differences between groups were analyzed by Student’s *t*-test or ANOVA in Graphpad prism 7.0 software. Pearson correlation analysis was used to assess the correlation between IGF2BP2 and NRP1 expression in BCa tumor tissues. *P* < 0.05 was regarded as statistically significant.

## Results

### IGF2BP2 was upregulated in BCa tissues and cells

**Fig. 1:**
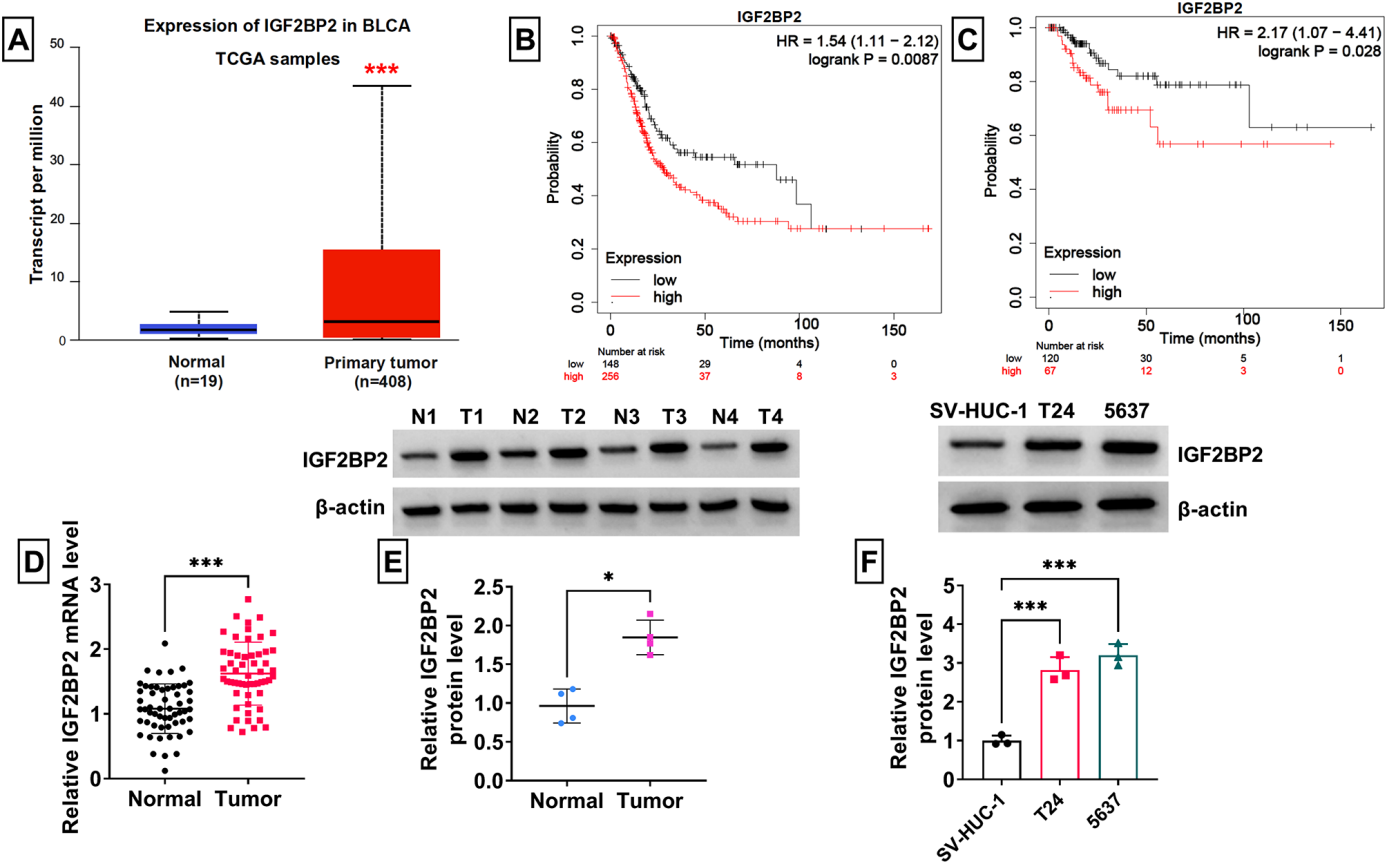
IGF2BP2 expression in BCa tissues and cells. (**A**) UALCAN database showed IGF2BP2 expression in BLCA tissues and normal tissues. (**B**-**C**) Kaplan-Meier plotter analyzed the relationship of IGF2BP2 expression with overall survival and relapse free survival (time to relapse) in BCa patients. (**D**) IGF2BP2 mRNA level was detected by RT-qPCR in BCa tumor tissues and adjacent normal tissues. (**E**-**F**) WB was used to analyze IGF2BP2 protein level in tissues and cells. *P < 0.05, ***P < 0.001

UALCAN database showed that IGF2BP2 was highly expressed in bladder urothelial carcinoma (BLCA) tissues (Fig. 1A). Besides, Kaplan-Meier plotter analysis also indicated that BCa patients with high IGF2BP2 expression had lower overall survival and shorter relapse free survival (time to relapse) (Fig. 1B-C). Through RT-qPCR and WB analysis, we confirmed that IGF2BP2 expression was higher in BCa tumor tissues than in normal tissues at the mRNA and protein levels (Fig. 1D-E). Compared to SV-HUC-1 cells, IGF2BP2 protein level also was increased in BC cell lines (T24 and 5637) (Fig. 1F).

### Downregulation of IGF2BP2 suppressed BCa cell growth and metastasis

**Fig. 2:**
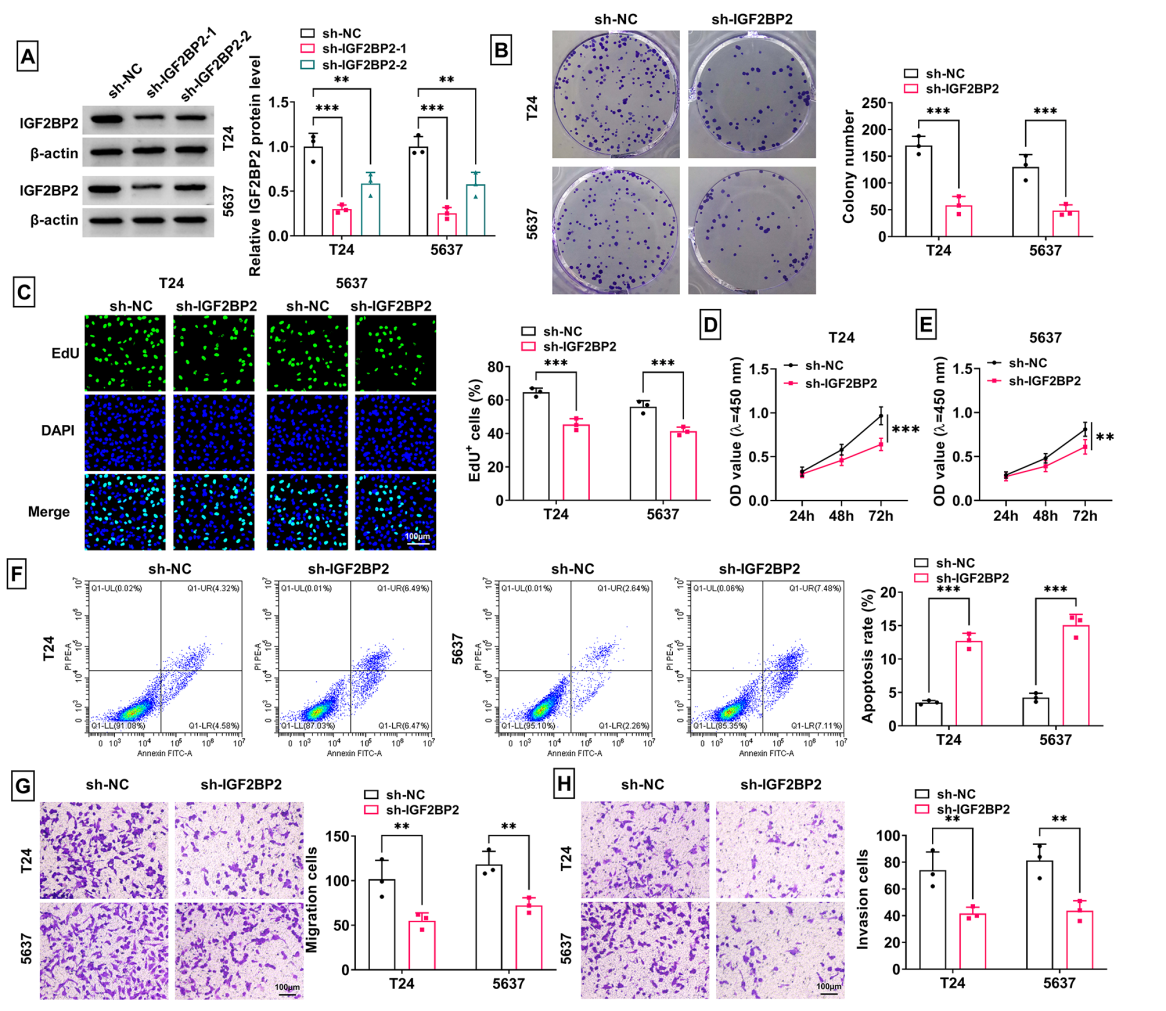
Effect of sh-IGF2BP2 on BCa cell growth and metastasis. (**A**) The transfection efficiency of sh-IGF2BP2-1/2 was confirmed by WB. (B-H) T24 and 5637 cells were transfected with sh-NC/sh-IGF2BP2. Colony formation assay (**B**), EdU assay (**C**), CCK8 assay (**D**-**E**), flow cytometry (**F**) and transwell assay (**G**-**H**) were performed to measure cell proliferation, apoptosis, migration and invasion. **P < 0.01, ***P < 0.001

Then, we assessed IGF2BP2 roles in BCa cell progression. After transfection, IGF2BP2 protein expression was significantly reduced, among which sh-IGF2BP2-1 had the best effect (Fig. 2A). Therefore, sh-IGF2BP2-1 (abbreviated as sh-IGF2BP2) was selected for subsequent study. As presented in Fig. 2B-F, IGF2BP2 knockdown inhibited the colony number, EdU^+^ cell rate and cell viability, while increased apoptosis rate of T24 and 5637 cells. Moreover, silencing of IGF2BP2 also repressed the migration and invasion of T24 and 5637 cells (Fig. 2G-H). Additionally, we measured the effect of IGF2BP2 overexpression on BCa progression. After transfection of OE-IGF2BP2, IGF2BP2 protein level was markedly enhanced in T24 and 5637 cells (Supplementary Fig. 1A). Overexpressed IGF2BP2 promoted colony numbers, EdU^+^ cell rate and cell viability (Supplementary Fig. 1B-E). Moreover, IGF2BP2 overexpression suppressed apoptosis rate, while enhanced migration and invasion of T24 and 5637 cells (Supplementary Fig. 1F-H).

### IGF2BP2 knockdown reduced BCa tumor growth

**Fig. 3:**
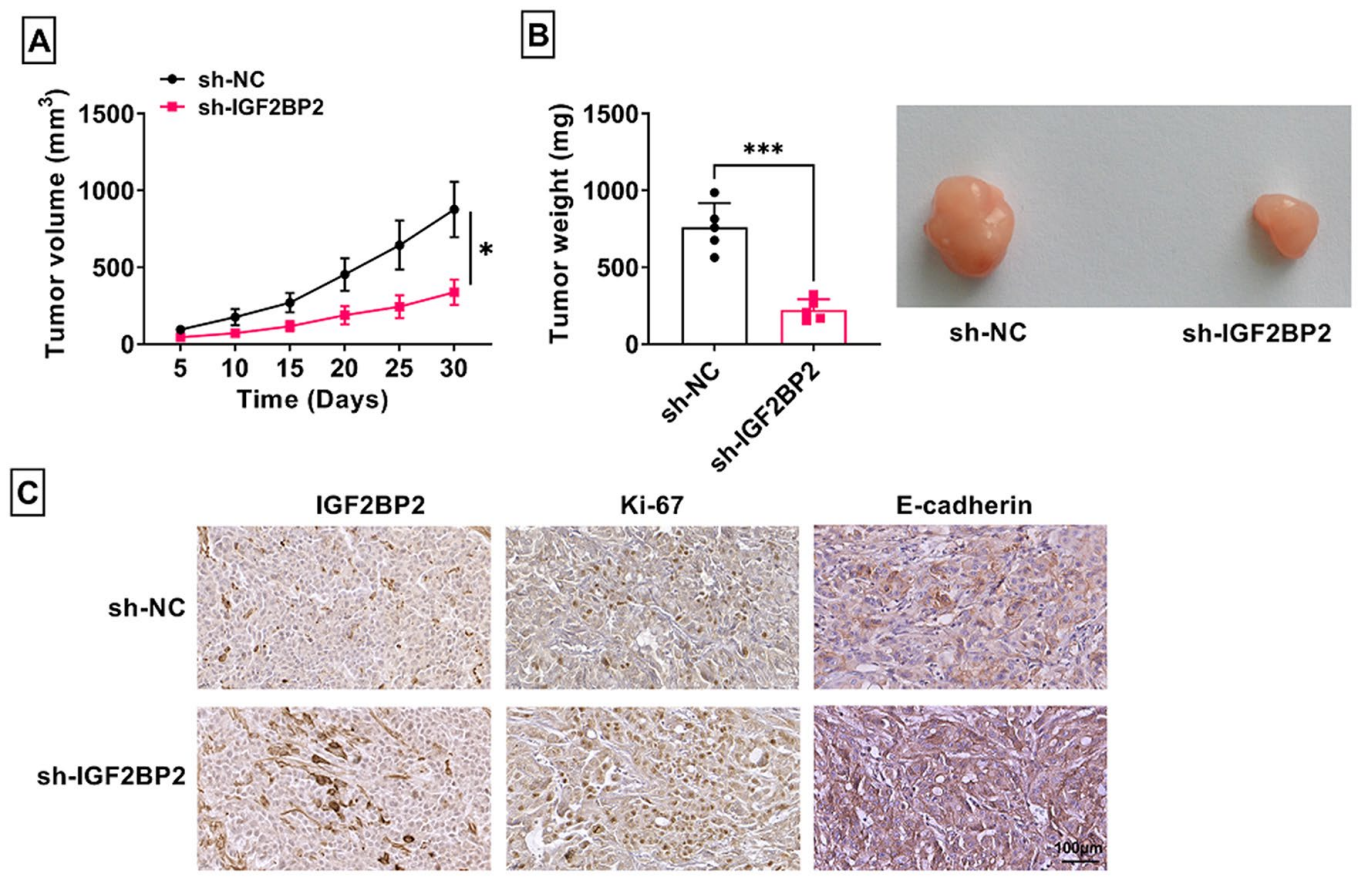
Effect of IGF2BP2 knockdown on BCa tumor growth. 5637 cells transfected with sh-NC/sh-IGF2BP2 were injected into nude mice. Tumor volume (**A**) and weight (**B**) were detected. (**C**) IHC staining was used to measure the positive cells of IGF2BP2, Ki-67 and E-cadherin in tumor tissues. *P < 0.05, ***P < 0.001

To further confirm the pro-tumor role of IGF2BP2 in BCa, we performed xenograft tumor experiments. The results suggested that tumor volume and weight were markedly reduced in sh-IGF2BP2 group (Fig. 3A-B). Also, the positive cells of IGF2BP2, proliferation marker Ki-67 and metastasis marker E-cadherin were decreased in the tumor tissues of sh-IGF2BP2 group (Fig. 3C).

### IGF2BP2 promoted recruitment and polarization of M2 macrophages

**Fig. 4:**
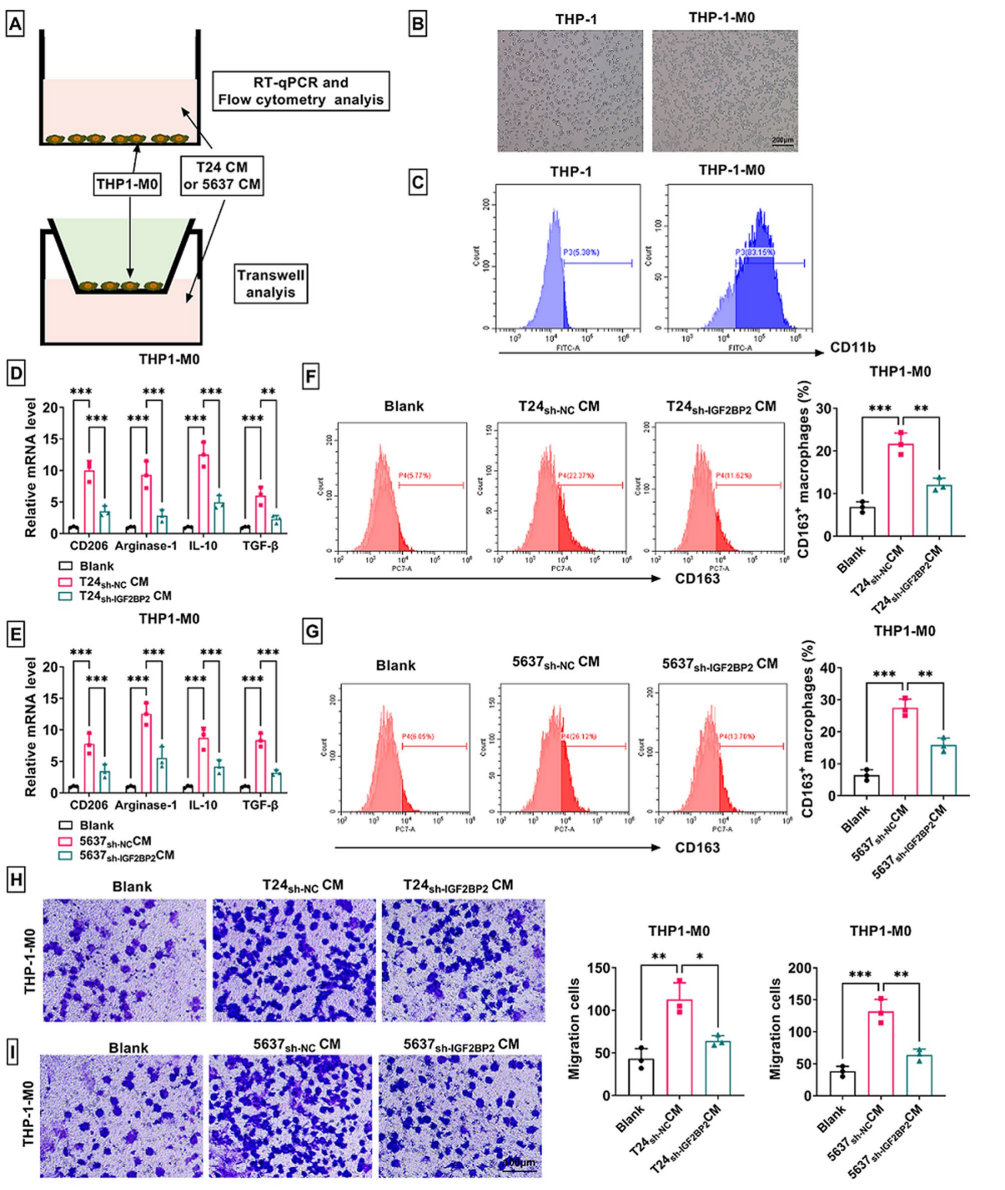
Effect of sh-IGF2BP2 on the recruitment and polarization of M2 macrophages. (**A**) A flow chart of the co-culture is shown. (**B**) The morphology of THP-1 and THP-1-M0 is shown. (**C**) Flow cytometry was used to measure CD11b level. (**D**-**E**) The mRNA levels of CD206, Arginase-1, IL-10 and TGF-β were examined by RT-qPCR. (**F**-**G**) CD163^+^ macrophage rate was analyzed by flow cytometry. (**H**-**I**) The migration of THP-1-M0 was detected by transwell assay. *P < 0.05, **P < 0.01, ***P < 0.001

To explore the effect of IGF2BP2 on the recruitment and polarization of M2 macrophages, THP1-M0 macrophages were cultured with the CM of T24 and 5637 cells transfected with sh-NC/sh-IGF2BP2 (Fig. 4A). The morphological diagrams of THP-1 cells and THP1-M0 macrophages are shown in Fig. 4B. Flow cytometry analysis showed that macrophage marker CD11b was observed in THP1-M0 macrophages (Fig. 4C), confirming the successful construction of macrophages. The mRNA levels of M2 macrophage markers (CD206, Arginase-1, IL-10 and TGF-β) were dramatically increased in THP1-M0 macrophages cultured with CM of sh-NC-transfected T24 and 5637 cells, and this effect was partially eliminated in macrophages incubated with the CM of sh-IGF2BP2-transfected cells (Fig. 4D-E). Besides, M2 macrophage marker CD163^+^ rate and migration ability in THP1-M0 macrophages induced by the CM of sh-NC-transfected T24 and 5637 cells were also significantly impaired in response to sh-IGF2BP2 (Fig. 4F-I).

### m6A-reader IGF2BP2 enhanced the stability of NRP1 mRNA

**Fig. 5:**
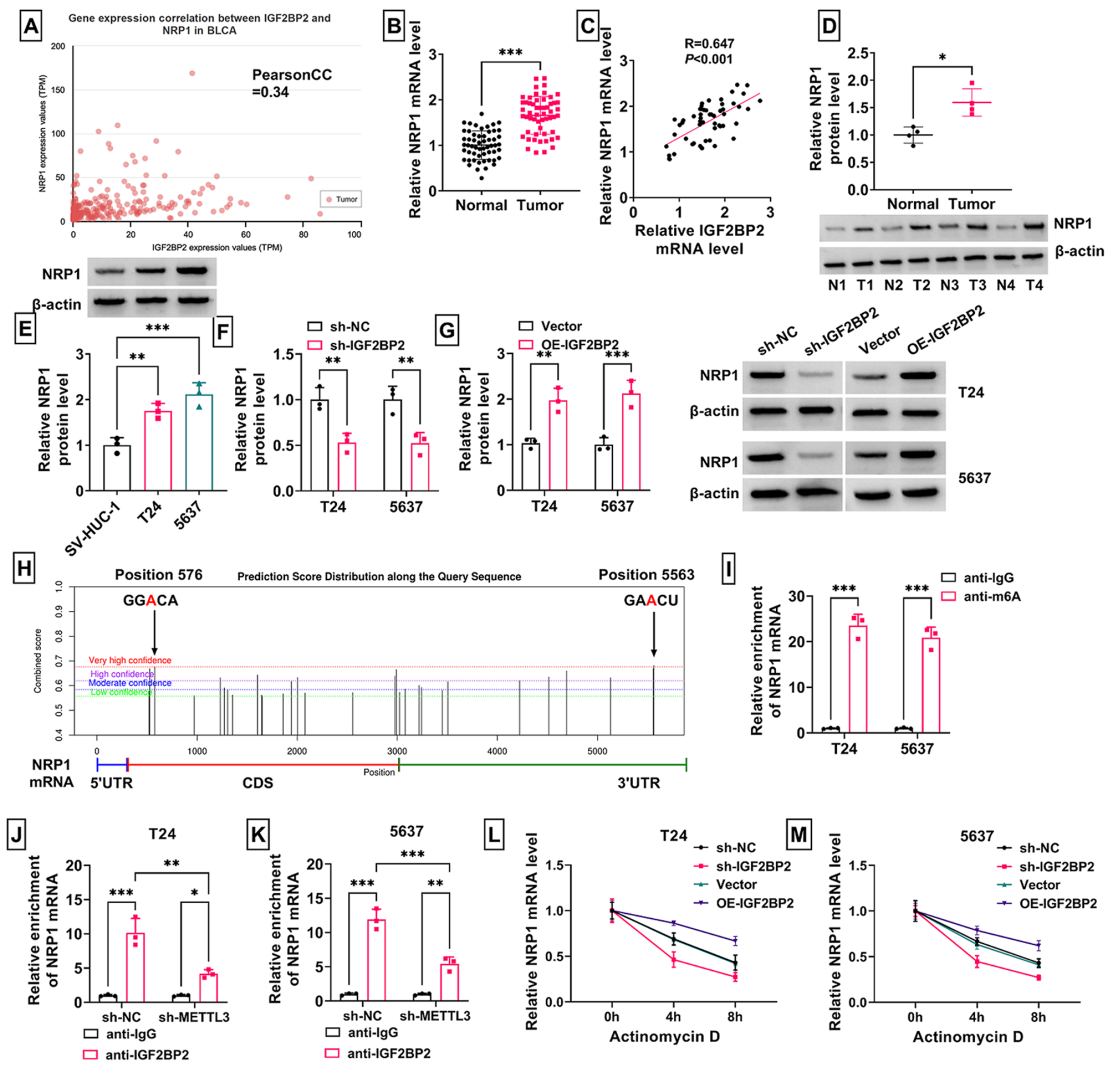
Effect of IGF2BP2 on the stability of NRP1 mRNA. (**A**) UALCAN database analyzed the correlation between IGF2BP2 and NRP1 expression in BLCA tissues. (**B**) NRP1 mRNA level was measured by RT-qPCR in BCa tumor tissues and adjacent normal tissues. (**C**) Pearson correlation analysis was used to detect the correlation between IGF2BP2 and NRP1 expression in BCa tumor tissues. (**D**-**E**) NRP1 protein level was tested by WB in BCa tumor tissues and cells. (**F**-**G**) NRP1 protein level in T24 and 5637 cells transfected with sh-NC/sh-IGF2BP2/Vector/OE-IGF2BP2 was examined by WB. (**H**) SRAMP predicted the m6A binding sites in NRP1 mRNA. (**I**) MeRIP assay was used to confirm the enrichment of NRP1 mRNA in anti-m6A. (**J**-**K**) RIP assay was used to detect the interaction between NRP1 and IGF2BP2 in T24 and 5637 cells transfected with sh-NC/sh-METTL3. (**L**-**M**) Actinomycin D was used to measure the effect of sh-IGF2BP2 or OE-IGF2BP2 on the stability of NRP1 mRNA. *P < 0.05, **P < 0.01, ***P < 0.001

UALCAN database suggested that the expression of IGF2BP2 and NRP1 in BLCA was positively correlated (Fig. 5A). In this, we detected high mRNA level of NRP1 in BCa tumor tissues and confirmed that NRP1 level was positively correlated with IGF2BP2 mRNA level (Fig. 5B-C). Besides, NRP1 protein level also was higher in BCa tumor tissues and cell lines (Fig. 5D-E). At the protein level, NRP1 expression could be suppressed by IGF2BP2 knockdown and enhanced by IGFBP2 overexpression (Fig. 5F-G). Through SRAMP prediction, we found that NRP1 mRNA had 2 m6A binding sites (Fig. 5H), which was further confirmed by MeRIP assay (Fig. 5I). To further verify that m6A modification was required for IGF2BP2 binding to NRP1 mRNA, we performed RIP experiments. The results showed that knockdown of m6A-writter METTL3 significantly reduced the enrichment of NRP1 mRNA binding to IGF2BP2 protein (Fig. 5J-K). Actinomycin D assay results indicated that IGF2BP2 knockdown suppressed the stability of NRP1 mRNA, while its overexpression had an opposite effect (Fig. 5L-M).

### NRP1 overexpression promoted BCa progression regulated by sh-IGF2BP2

**Fig. 6:**
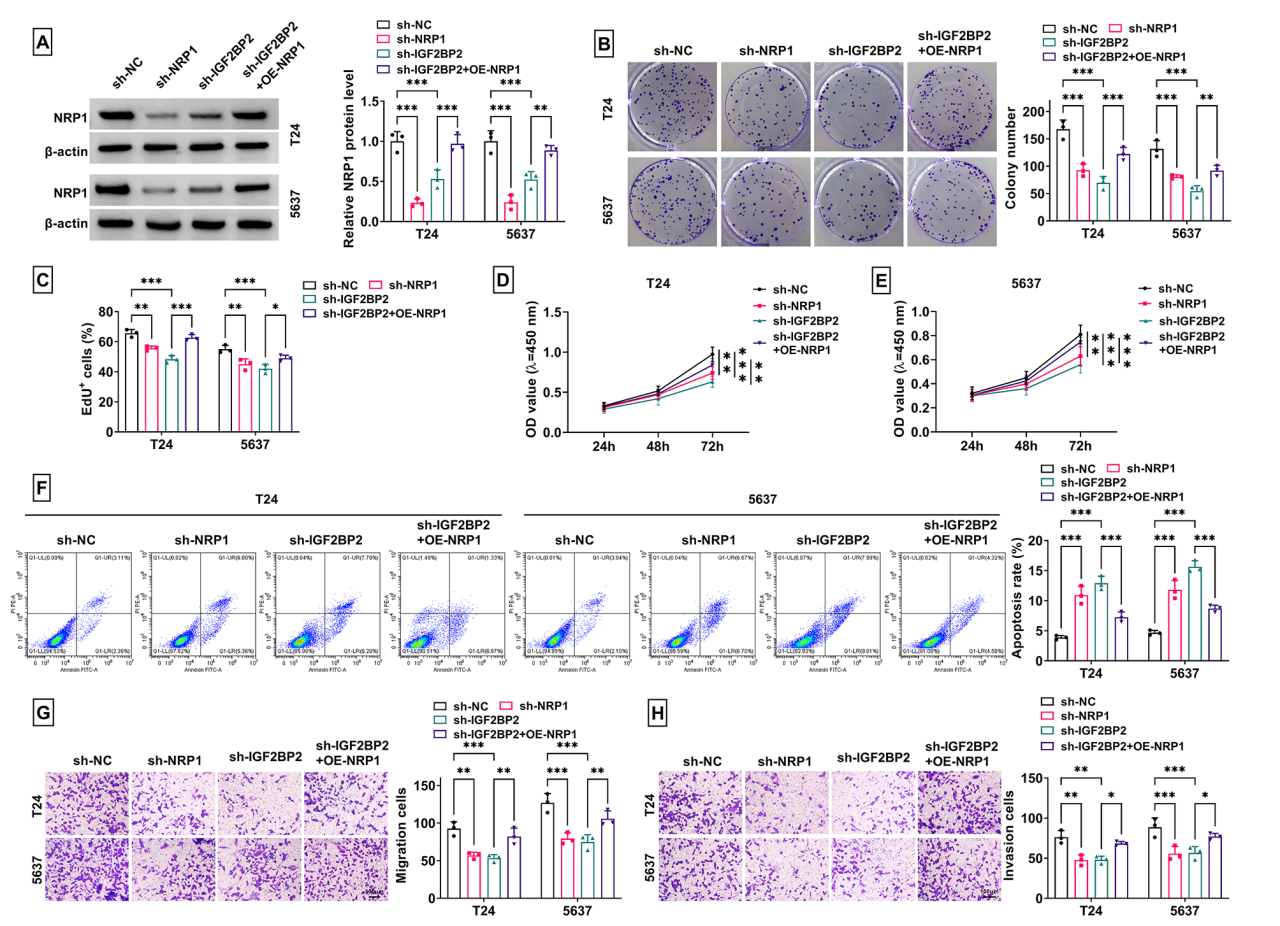
Effect of sh-NRP1, OE-NRP1 and sh-IGF2BP2 on BCa progression. T24 and 5637 cells were transfected with sh-NC/sh-NRP1/sh-IGF2BP2/sh-IGF2BP2 + OE-NRP1. (**A**) NRP1 protein level was measured by WB. Cell proliferation, apoptosis, migration and invasion were examined by colony formation assay (**B**), EdU assay (**C**), CCK8 assay (**D**-**E**), flow cytometry (**F**) and transwell assay (**G**-**H**). *P < 0.05, **P < 0.01, ***P < 0.001

Further analysis was performed to explore NRP1 roles in BCa progression and whether IGF2BP2 regulated NRP1 to mediate BCa progression. The detection of NRP1 protein level confirmed that NRP1 expression could be reduced by sh-NRP1 and sh-IGF2BP2, and OE-NRP1 markedly enhanced NRP1 expression in sh-IGF2BP2-transfected T24 and 5637 cells (Fig. 6A). NRP1 knockdown reduced colony number, EdU^+^ cell rate and cell viability, while enhanced apoptosis rate in T24 and 5637 cells. And the regulation of sh-IGF2BP2 on BCa cell proliferation and apoptosis could be reversed by NRP1 overexpression (Fig. 6B-F). NRP1 downregulation also inhibited the migration and invasion of T24 and 5637 cells, and the suppressive effect of sh-IGF2BP2 on cell migration and invasion could be eliminated by NRP1 overexpression (Fig. 6G-H).

### NRP1 overexpression overturned the effect of IGF2BP2 knockdown on M2 macrophage polarization

**Fig. 7:**
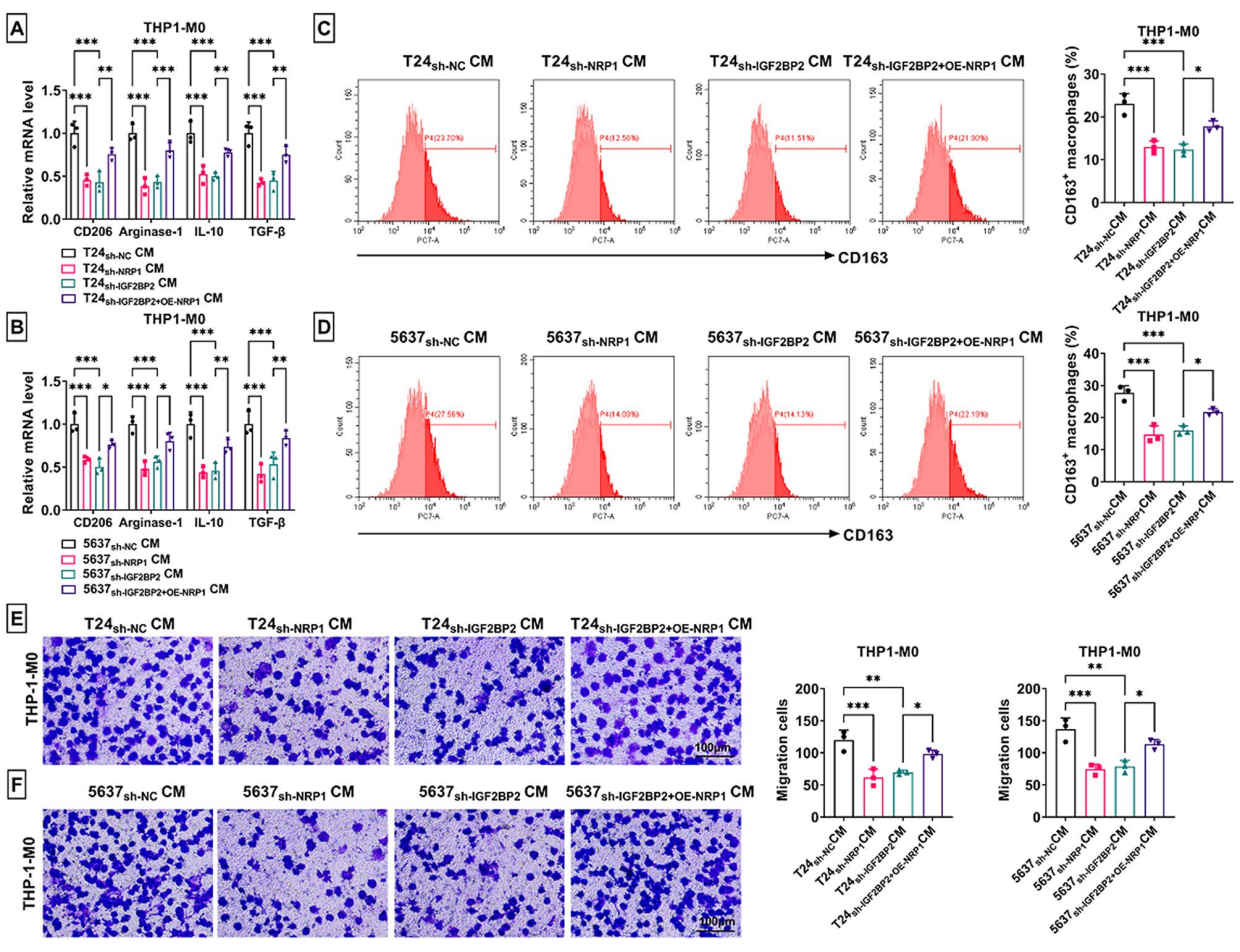
Effect of sh-NRP1, OE-NRP1 and sh-IGF2BP2 on M2 macrophage polarization. (**A**-**B**) RT-qPCR was used to measure the mRNA levels of CD206, Arginase-1, IL-10 and TGF-β. (**C**-**D**) Flow cytometry was performed to examine CD163^+^ macrophage rate. (**E**-**F**) Transwell assay was employed to detect cell migration. *P < 0.05, **P < 0.01, ***P < 0.001

Then, THP1-M0 macrophages were cultured with the CM of T24 and 5637 cells transfected with sh-NC/sh-NRP1/sh-IGF2BP2/sh-IGF2BP2 + OE-NRP1. The mRNA levels of CD206, Arginase-1, IL-10 and TGF-β were reduced in THP1-M0 macrophages cultured with CM of T24 and 5637 cells transfected with sh-NRP1 or sh-IGF2BP2, while OE-NRP1 partially abolished the effect of sh-IGF2BP2 (Fig. 7A-B). Also, CD163^+^ rate and migration ability of THP1-M0 macrophages cultured with CM of T24 and 5637 cells transfected with sh-NRP1 or sh-IGF2BP2 were reduced, while the effect of sh-IGF2BP2 also partially eliminated by OE-NRP1 (Fig. 7C-F).

## Discussion

The incidence of BCa ranks in the forefront of urinary system malignant tumors, which poses a great threat to the lives of patients [25]. Macrophages can be induced to different polarization states depending on the different microenvironment they receive to perform a variety of functions, such as inflammation, immune regulation, and tumorigenesis [26, 27]. The function of tumor-associated macrophages is closely related to the tumor microenvironment, and M2 macrophages have been shown to promote tumorigenesis [28, 29]. Studies have shown that promoting M2 macrophage polarization may accelerate the malignant progression of BCa [6, 7]. Therefore, revealing the molecular mechanisms that affect M2 macrophage polarization and BCa progression is important for BCa treatment. In this, we found that IGF2BP2 promoted NRP1 mRNA stability to facilitate M2 macrophage polarization and BCa progression, providing new ideas for BCa treatment.

m6A methylation is the most important chemical modification of RNA and plays a key role in tumor development and drug resistance [30]. IGF2BP2 is a key gene in m6A signaling, and its abnormal expression has been shown to be associated with tumorigenesis [31]. It had been reported that IGF2BP2 overexpression aggravated cell proliferation in colorectal cancer [32] and hepatocellular carcinoma [33]. Importantly, IGF2BP2 could stabilize TGFBR1 expression, thus mediating the promotion of M2 macrophage polarization and lung cancer malignant process [34]. Also, the accumulation of IGF2BP2 could activate the RhoA/Rock pathway, which in turn promoted M2 macrophage polarization and accelerated colorectal cancer progression [35]. These evidences suggest that IGF2BP2 may play an active role in M2 macrophage polarization and tumorigenesis. Although previous studies have shown that IGF2BP2 expression may be related to BCa patients’ prognosis and can mediate BCa progression [14, 15], its specific role has not been revealed. In this, we determined that IGF2BP2 was highly expressed in BCa, and its knockdown could inhibit BCa cell growth and metastasis, as well as tumor growth. In addition, we confirmed that silencing of IGF2BP2 reduced M2 macrophage polarization. These results provide evidence for a deeper understanding of the oncogenic role of IGF2BP2 in BCa.

Previous study had suggested that IGF2BP2 elevated FLT4 stability via m6A modification, thus accelerating angiogenesis and metastasis of lung adenocarcinoma cells [36]. Also, IGF2BP3 promoted pancreatic carcinoma cell proliferation and migration by improving B3GNT6 mRNA stability [37]. Here, we pointed out that IGF2BP2, as an m6A reader, recognized and bound to the m6A modified region of NRP1 transcript to enhance NRP1 mRNA stability. NRP1 is commonly expressed in endothelial cells and some tumor cells, and plays an important function in angiogenesis and tumorigenesis [38, 39]. In many cancers, NRP1 serves as tumor promoter to participate in development of many cancers, including gastric cancer [40] and lung cancer [41]. It has been reported that NRP1 is overexpressed in BCa and promotes the proliferation, angiogenesis and metastasis in BCa cells [42]. Similarly, the same results were obtained in our study. Consistent with previous data [22, 23], we also proved the promotion effect of NRP1 on M2 macrophage polarization. Furthermore, overexpression of NRP1 eliminated the inhibitory effect of IGF2BP2 silencing on M2 macrophage polarization and BCa cell functions, which further confirmed that IGF2BP2 indeed stabilized NRP1 mRNA to accelerate M2 macrophage polarization and BCa progression. Of course, this study has certain limitations. BCa tissue is composed of cancer cells and immune-infiltrating cells. This study confirmed that IGF2BP2 is highly expressed in BCa cells. However, we are not sure whether IGF2BP2 is also highly expressed in immune infiltrating cells, which needs to be further explored in future studies.

In conclusion, our study suggests a novel regulatory axis that mediates BCa progression. Our data indicated that IGF2BP2 could improve NRP1 mRNA stability, thereby promoting M2 macrophage polarization and BCa progression. These findings provide a new direction for BCa treatment and have important clinical implications.

### Electronic supplementary material

Below is the link to the electronic supplementary material.

Supplementary Material 1

Supplementary Material 2

## Data Availability

The analyzed data sets generated during the present study are available from the corresponding author on reasonable request.
