# Supplementary material for: m6A reader IGF2BP2 promotes M2 macrophage polarization and malignant biological behavior of bladder cancer by stabilizing NRP1 mRNA expression
Source: BMC Urol. 2024 Jul 16;24:147. doi: 10.1186/s12894-024-01534-4 (PMC11251312; doi:10.1186/s12894-024-01534-4)
Supplement: Supplementary file 1 — Supplementary Material 1 [file 12894_2024_1534_MOESM1_ESM.pdf]

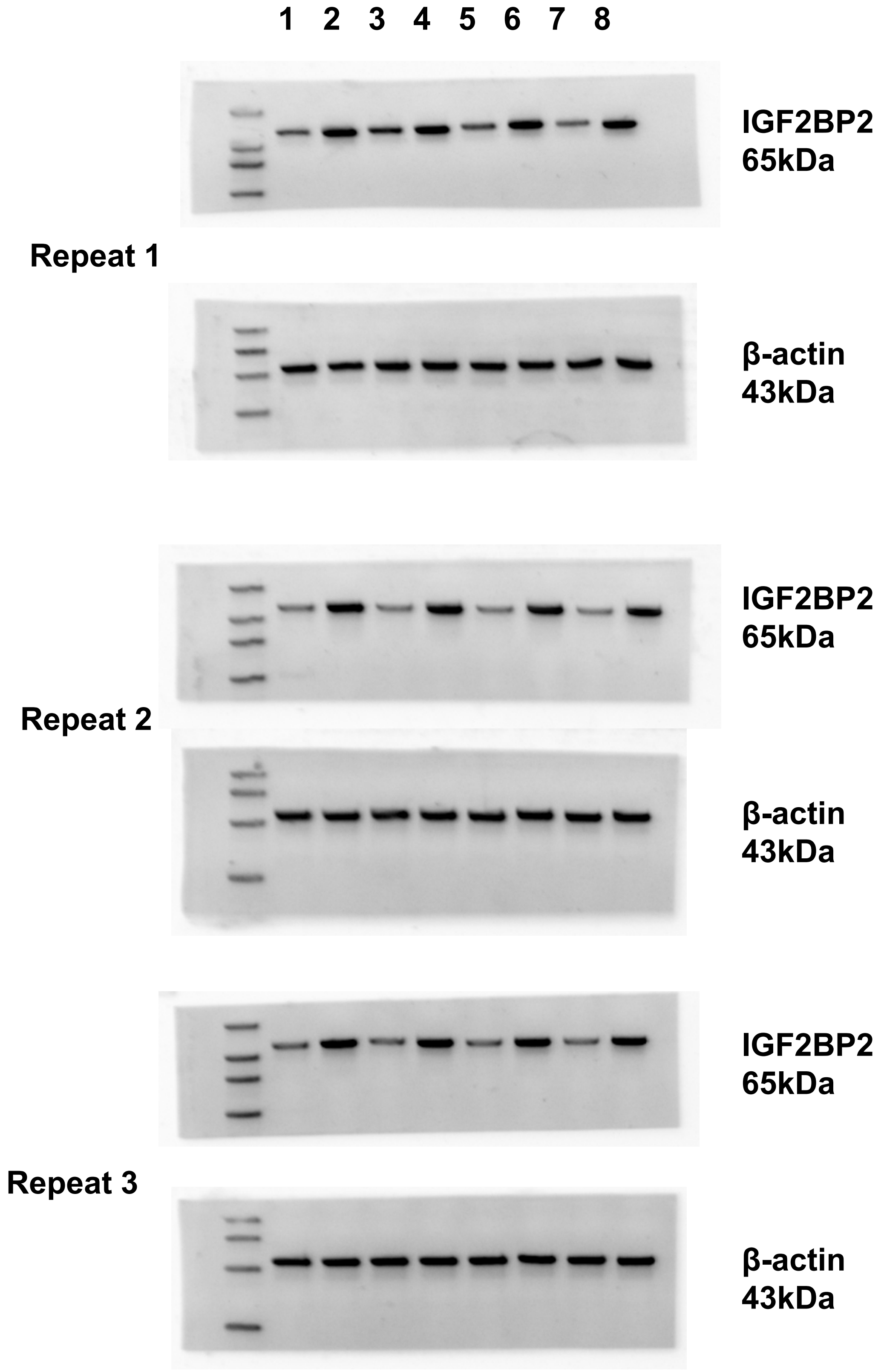

1 N1
2 T1
3 N2
4 T2
5 N3
6 T3
7 N4
8 T4

Fig 1E

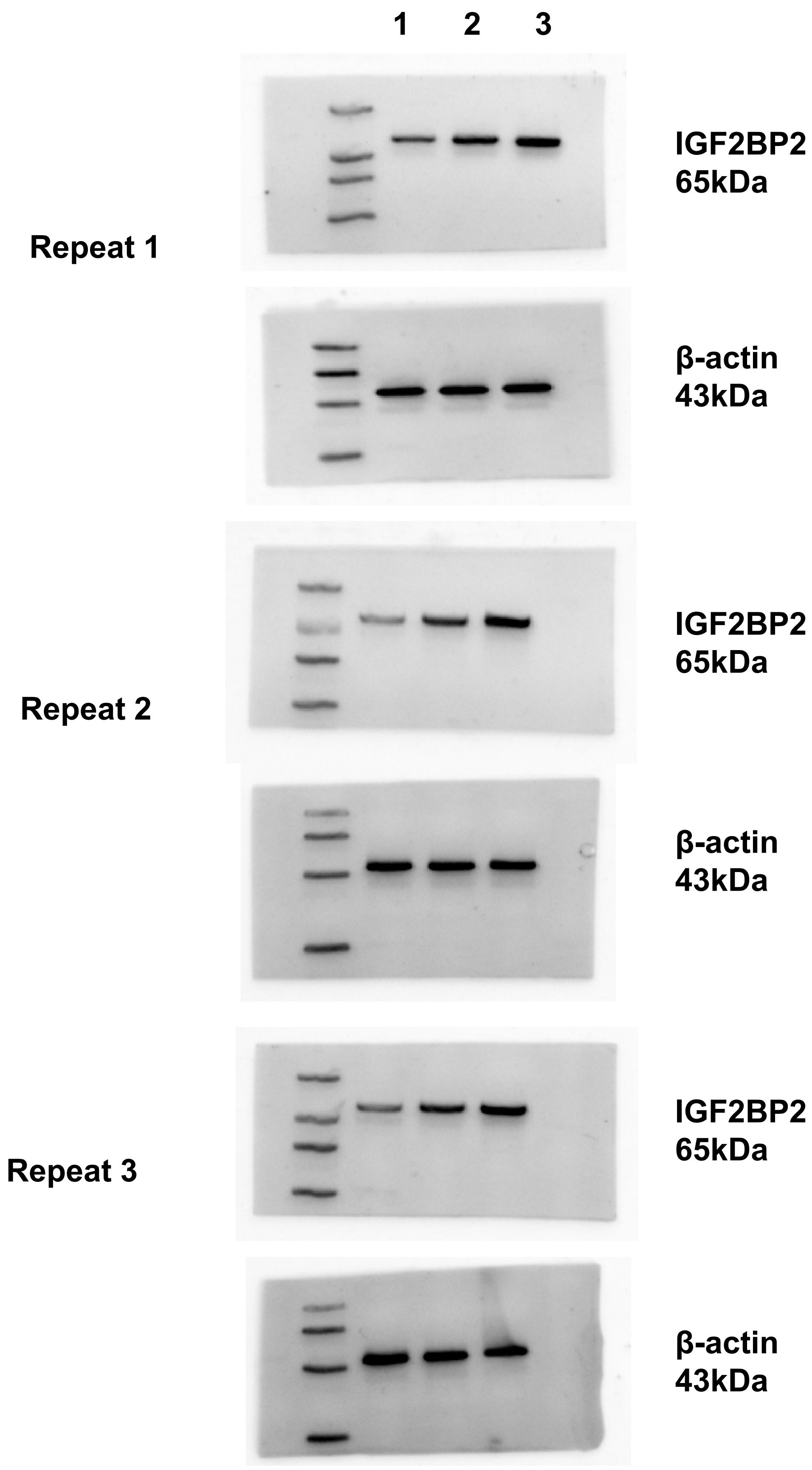

1 SV-HUC-1
2 T24
3 5637

Fig 1F

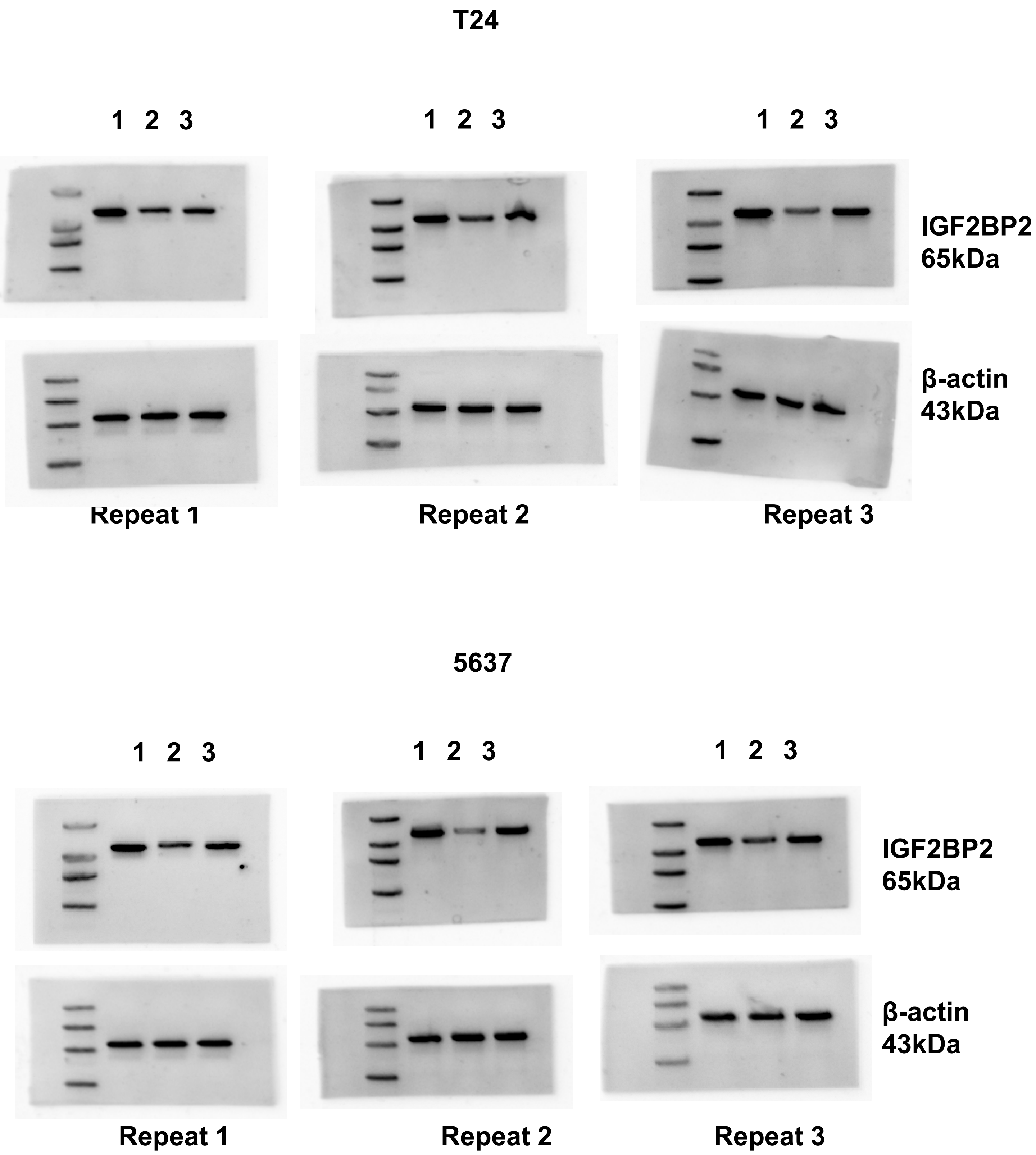

1 sh-NC

2 sh-IGF2BP2-1

Fig 2A 3 sh-IGF2BP2-2

1 2 3 4 5 6 7 8

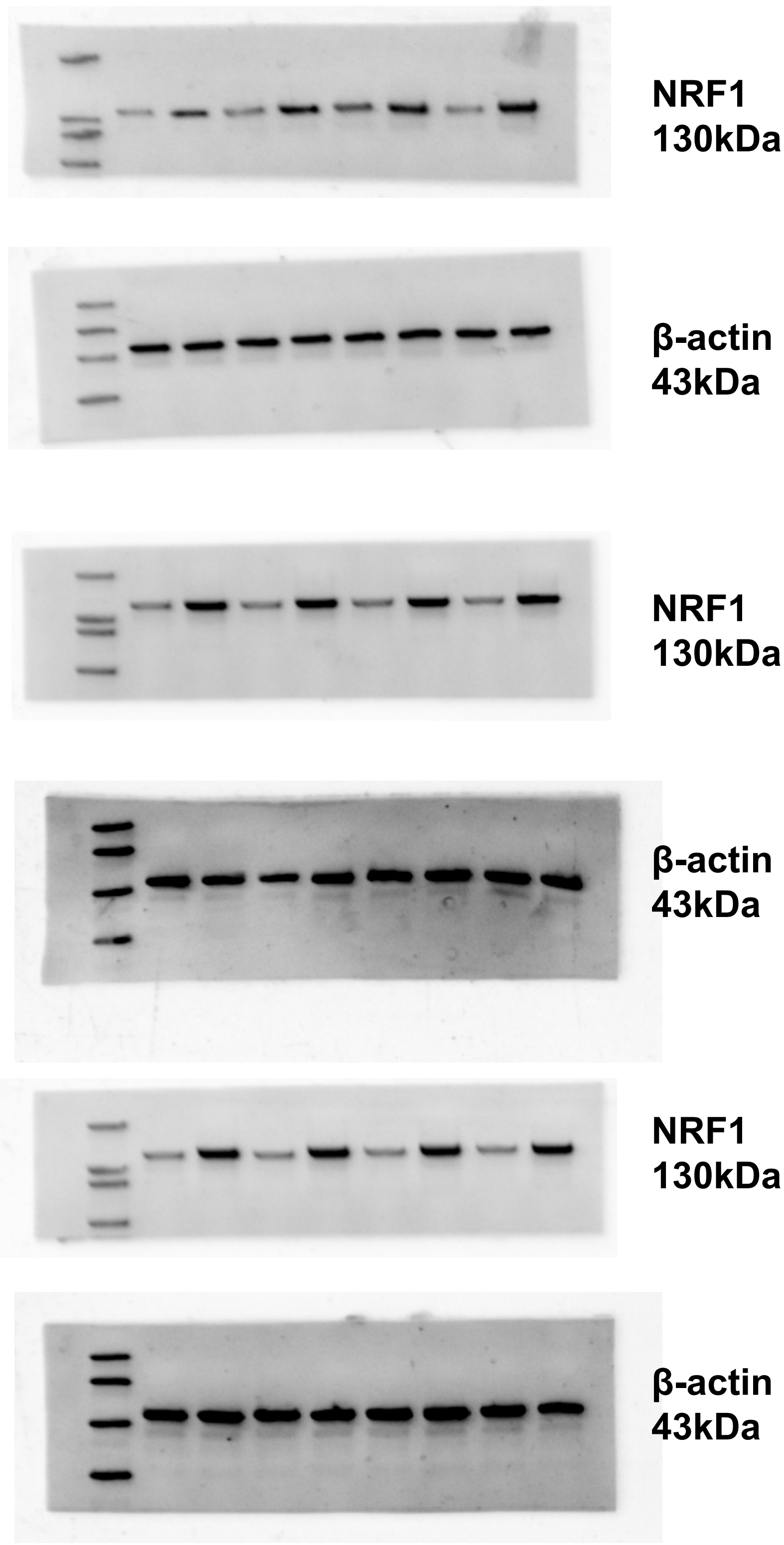

NRF1
130kDa

Repeat 1

β-actin
43kDa

NRF1
130kDa

Repeat 2

β-actin
43kDa

NRF1
130kDa

Repeat 3

β-actin
43kDa

1 N1
2 T1
3 N2
4 T2
5 N3
6 T3
7 N4
8 T4

Fig 5D

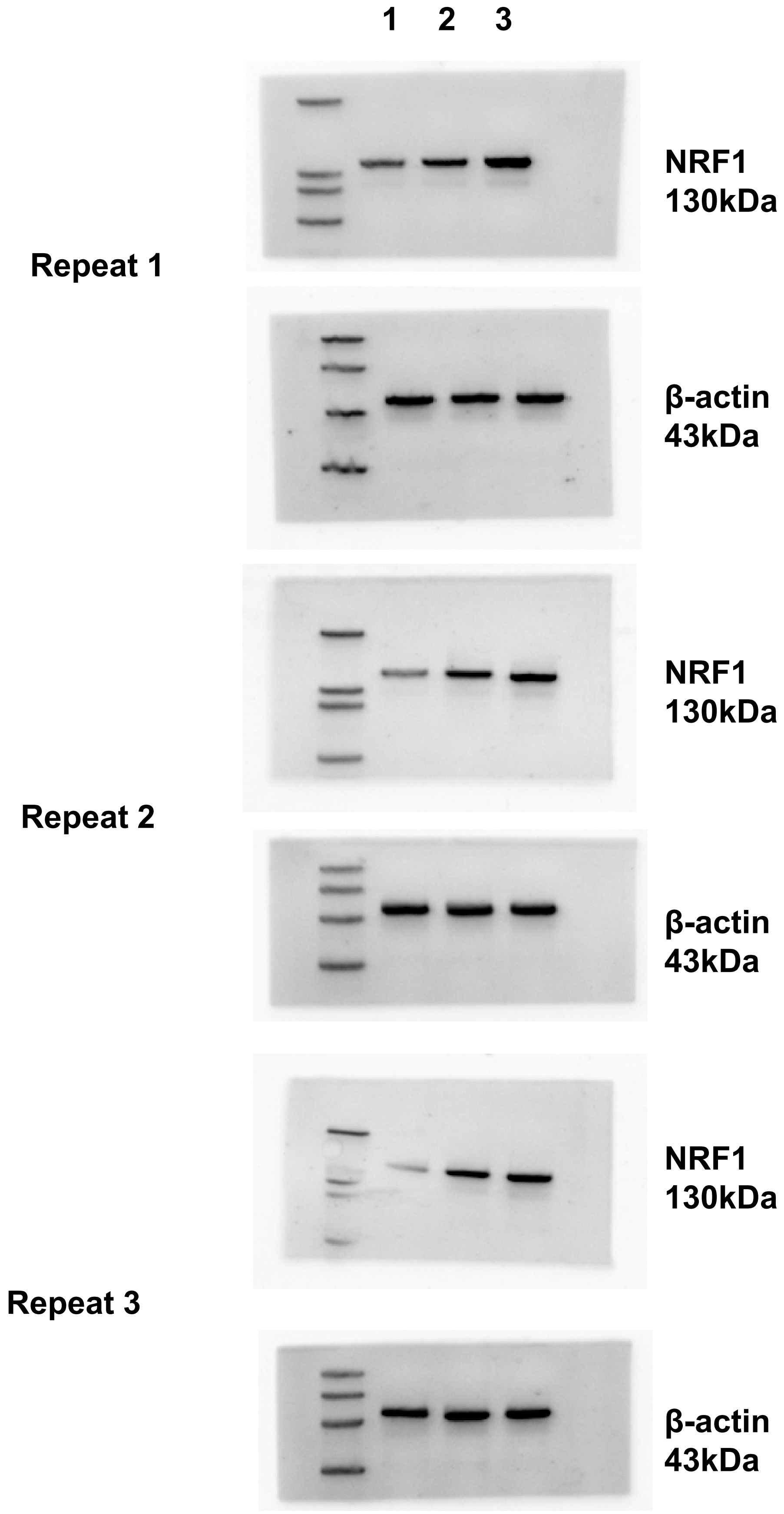

1 SV-HUC-1
2 T24
3 5637

Fig 5E

T24

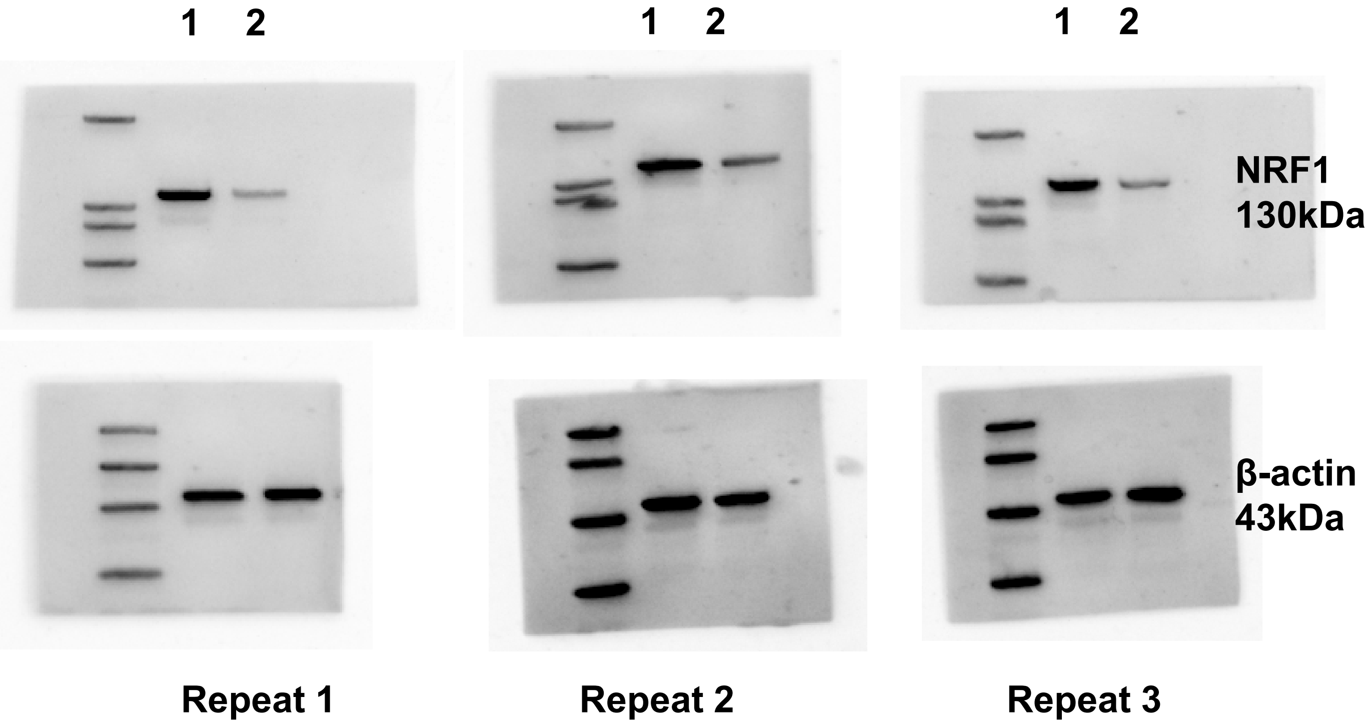

5637

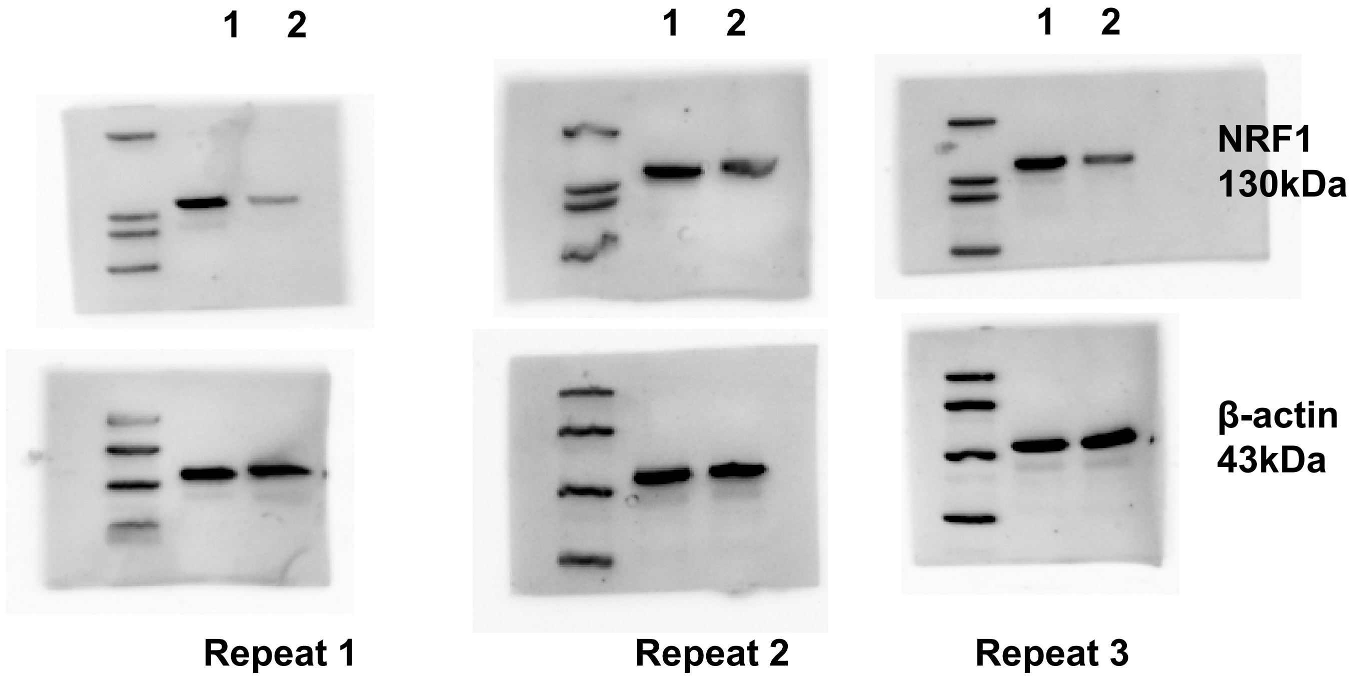

1 sh-NC

2 sh-IGF2BP2

Fig 5F

T24

1 2 1 2 1 2

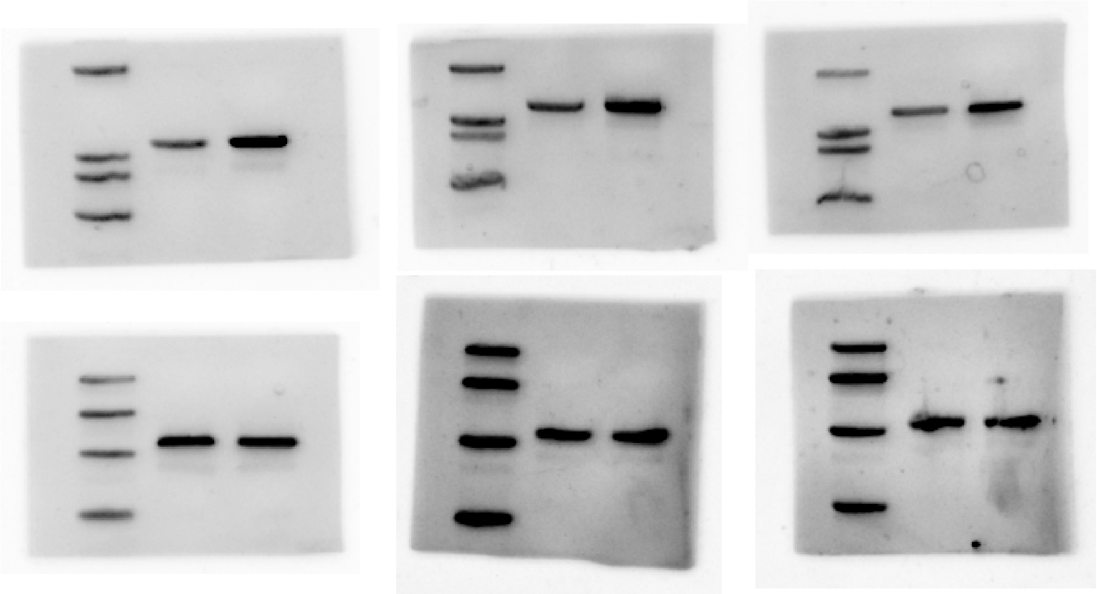

NRF1
130kDa

β-actin
43kDa

Repeat 1 Repeat 2 Repeat 3

5637

1 2 1 2 1 2

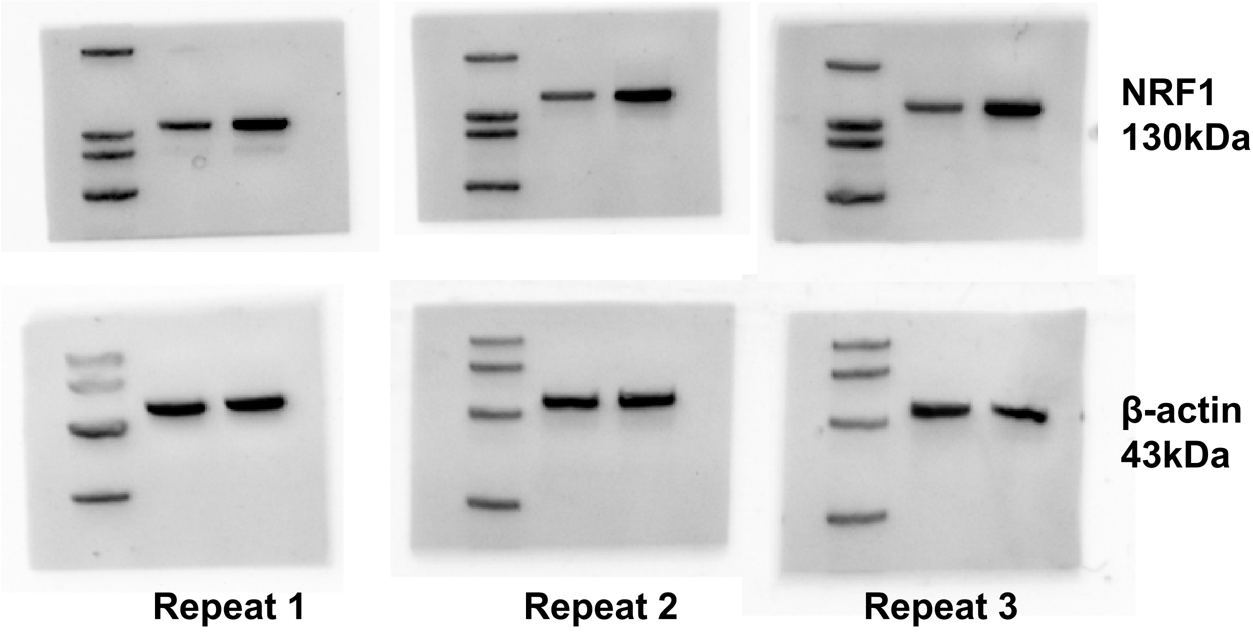

NRF1
130kDa

β-actin
43kDa

Repeat 1 Repeat 2 Repeat 3

Fig 5G

1 Vector
2 OE-IGF2BP2

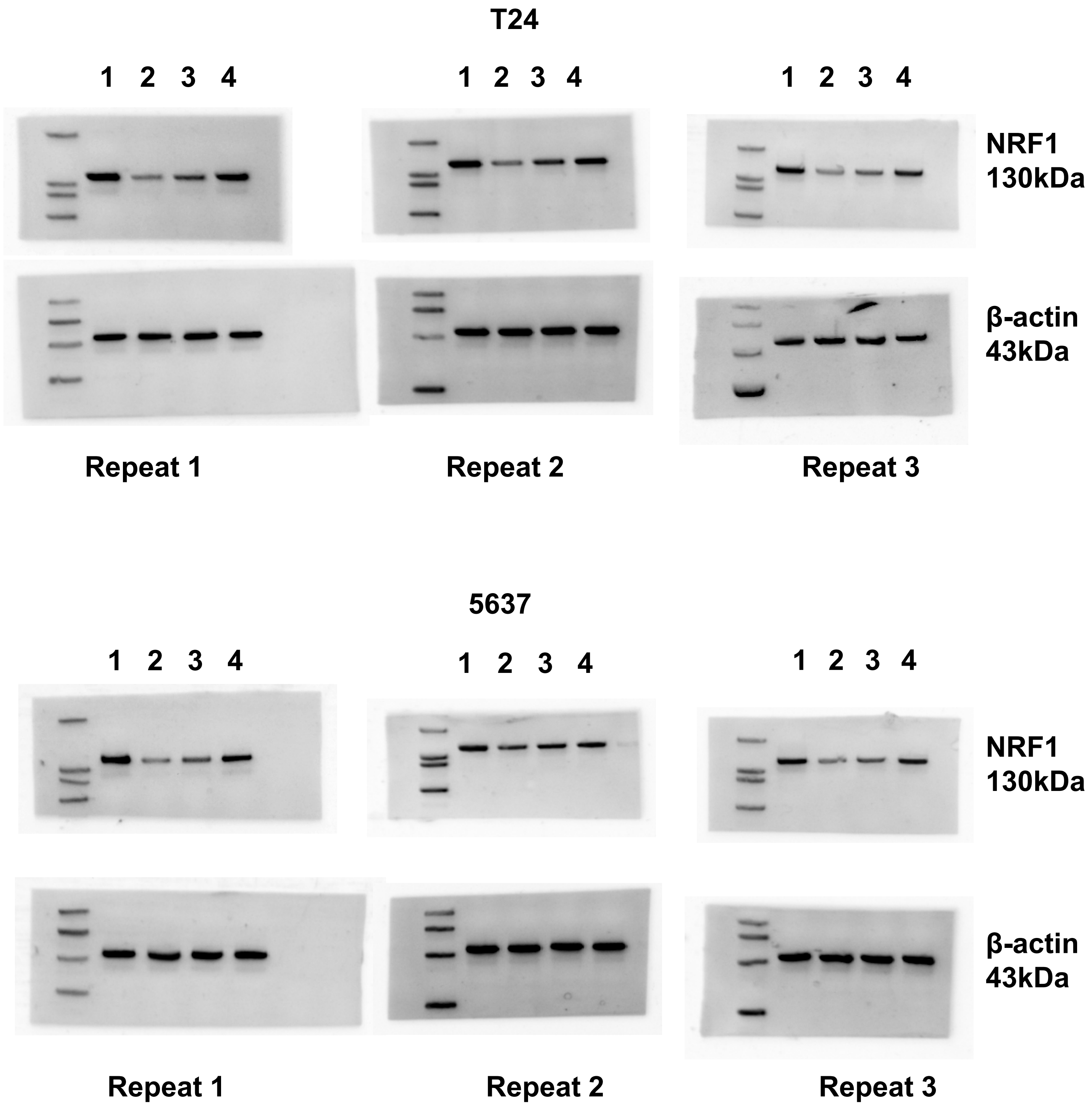

Fig 6A

1 sh-NC

2 sh-NRF1

3 sh-IGF2BP2

4 sh-IGF2BP2+OE-NRF1

Protein Marker

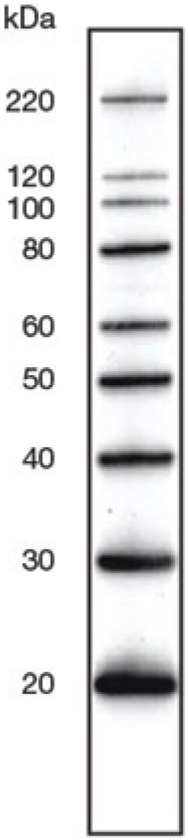

- **The samples derive from the same experiment and that gels/blots were processed in parallel.**
