# Supplementary material for: m6A reader IGF2BP2 promotes M2 macrophage polarization and malignant biological behavior of bladder cancer by stabilizing NRP1 mRNA expression
Source: BMC Urol. 2024 Jul 16;24:147. doi: 10.1186/s12894-024-01534-4 (PMC11251312; doi:10.1186/s12894-024-01534-4)
Supplement: Supplementary file 2 — Supplementary Material 2 [file 12894_2024_1534_MOESM2_ESM.docx]

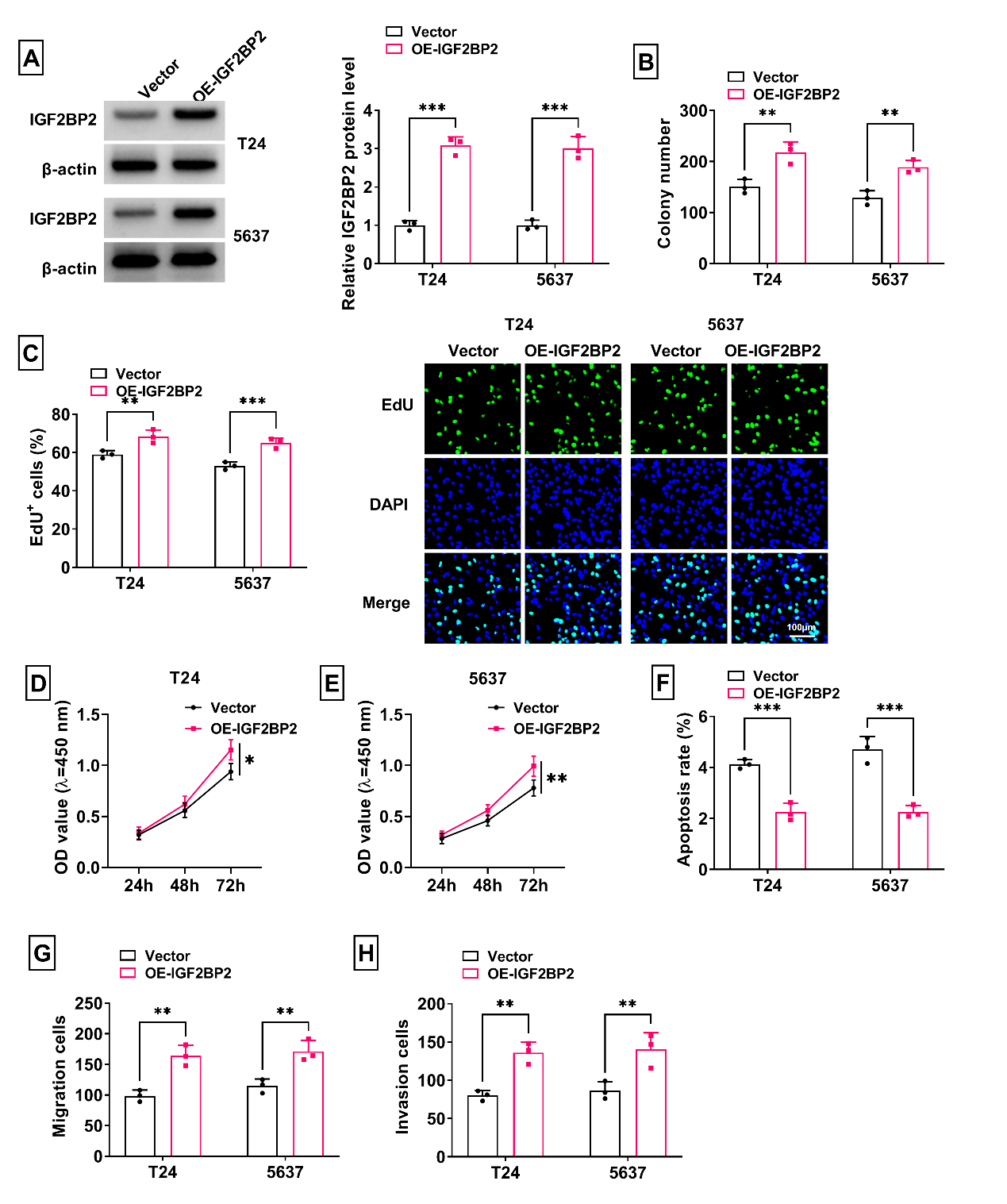
**Supplementary Fig. 1 Effect of OE-IGF2BP2 on BCa progression.** T24 and 5637 cells were transfected with Vector/OE-IGF2BP2. (A) IGF2BP2 protein level was tested by WB. Cell proliferation, apoptosis, migration and invasion were assessed using colony formation assay (B), EdU assay (C), CCK8 assay (D-E), flow cytometry (F) and transwell assay (G-H). **P*<0.05, ***P*<0.01, ****P*<0.001.
